# Lenvatinib after progression on pemigatinib and futibatinib in FGFR2 fusion-positive biliary tract cancer with an acquired kinase point mutation

**DOI:** 10.1093/oncolo/oyaf322

**Published:** 2025-10-01

**Authors:** Fabian Kleinhenz, Nicole Pfarr, Lisa Steinhelfer, Alisa M Lörsch, Henriette Bendz, Mathias Friedrich, Lea Liesenfeld, Melissa Barroux, Carlo Maurer, Patrick Wenzel, Angelika Kestler, Mai-Lan Koppermann, Carolin Mogler, Stephan Spahn, Anna L Illert, Roland M Schmid, Michael Bitzer, Sebastian Lange

**Affiliations:** Clinical Department of Internal Medicine II, TUM School of Medicine and Health, TUM University Hospital, Munich 81675, Germany; Institute of Pathology, TUM School of Medicine and Health, Technical University of Munich, Munich 81675, Germany; Center for Personalized Medicine, TUM School of Medicine and Health, TUM University Hospital, Munich 81675, Germany; Department of Radiology, TUM School of Medicine and Health, TUM University Hospital, Munich 81675, Germany; Center for Personalized Medicine, TUM School of Medicine and Health, TUM University Hospital, Munich 81675, Germany; Clinical Department of Internal Medicine III, TUM School of Medicine and Health, TUM University Hospital, Munich 81675, Germany; Bavarian Cancer Research Center (BZKF), Munich 81675, Germany; Clinical Department of Internal Medicine II, TUM School of Medicine and Health, TUM University Hospital, Munich 81675, Germany; Clinical Department of Internal Medicine II, TUM School of Medicine and Health, TUM University Hospital, Munich 81675, Germany; Clinical Department of Internal Medicine II, TUM School of Medicine and Health, TUM University Hospital, Munich 81675, Germany; Clinical Department of Internal Medicine II, TUM School of Medicine and Health, TUM University Hospital, Munich 81675, Germany; Clinical Department of Internal Medicine II, TUM School of Medicine and Health, TUM University Hospital, Munich 81675, Germany; Clinical Department of Internal Medicine II, TUM School of Medicine and Health, TUM University Hospital, Munich 81675, Germany; Department of Internal Medicine I, University Hospital Ulm, Ulm 89081, Germany; Institute of Pathology, TUM School of Medicine and Health, Technical University of Munich, Munich 81675, Germany; Institute of Pathology, TUM School of Medicine and Health, Technical University of Munich, Munich 81675, Germany; Center for Personalized Medicine, TUM School of Medicine and Health, TUM University Hospital, Munich 81675, Germany; Department of Internal Medicine I, University Hospital, Eberhard-Karls University, 72076 Tuebingen, Germany; Center for Personalized Medicine, Eberhard-Karls University, Tuebingen 72076, Germany; Center for Personalized Medicine, TUM School of Medicine and Health, TUM University Hospital, Munich 81675, Germany; Clinical Department of Internal Medicine III, TUM School of Medicine and Health, TUM University Hospital, Munich 81675, Germany; Bavarian Cancer Research Center (BZKF), Munich 81675, Germany; Department of Hematology and Medical Oncology, University Medical Center Göttingen, Göttingen 37075, Germany; Clinical Department of Internal Medicine II, TUM School of Medicine and Health, TUM University Hospital, Munich 81675, Germany; Department of Internal Medicine I, University Hospital, Eberhard-Karls University, 72076 Tuebingen, Germany; Center for Personalized Medicine, Eberhard-Karls University, Tuebingen 72076, Germany; Clinical Department of Internal Medicine II, TUM School of Medicine and Health, TUM University Hospital, Munich 81675, Germany; Center for Personalized Medicine, TUM School of Medicine and Health, TUM University Hospital, Munich 81675, Germany; Bavarian Cancer Research Center (BZKF), Munich 81675, Germany

**Keywords:** BTC, CCA, FGFR alteration, FGFR2-fusion gene, TKIs, Lenvatinib

## Abstract

Biliary tract cancers (BTC) represent a heterogeneous group of malignancies with a poor prognosis and rising incidence. Oncogenic *FGFR2* fusions are one of several actionable molecular alterations. In this context, selective FGFR tyrosine kinase inhibitors have demonstrated promising and durable response rates and are now approved and included in clinical guidelines. However, secondary kinase mutations frequently arise over time, leading to resistance against these drugs. We present the case of a 41-year-old male patient with metastatic BTC who underwent molecular analysis after disease progression to various established chemotherapy combinations. Testing identified an oncogenic *FGFR2* fusion (*FGFR2::BICC1*). The patient was treated with pemigatinib for 14 months. Upon disease progression, the resistance-associated *FGFR2* p. E565A variant was detected in a follow-up biopsy. Treatment was switched to futibatinib, resulting in rapid disease progression. Lacking other therapeutic options, the patient was treated with lenvatinib, supported by previously published data suggesting a potential benefit in similar settings. The treatment was well tolerated, with only a mild increase in transaminases, and the patient remained on treatment with noteworthy effects for 15 months to date. With a growing incidence of BTC and growing use of targeted therapies for FGFR2 alterations, the emergence of secondary resistance-causing point mutations following treatment with approved inhibitors is becoming increasingly challenging. Beyond selective inhibitors, lenvatinib may represent a viable therapeutic option.

Key Points

- FGFR2 fusion-positive biliary tract cancers (BTC) can be treated with FGFR-directed therapeutics. However, secondary resistance mutations occur over time, limiting the long-term clinical efficacy of these therapies.
- We present the case of a patient with FGFR2 fusion-positive BTC previously treated with all available lines of chemotherapy as well as both approved FGFR inhibitors, pemigatinib and futibatinib, whose tumor was found to have developed a p. E565A resistance mutation in the molecular brake domain. The tumor responded to the multikinase inhibitor lenvatinib for a further 15 months.
- This case provides evidence that lenvatinib might be a valid option for patients with FGFR2 fusion-positive BTC who have exhausted other available therapies.

## Introduction

Biliary tract cancer (BTC) is a rare but aggressive malignancy with a poor prognosis.^1^ Within this group of tumors, which primarily includes cholangiocarcinoma, its intrahepatic subtype (iCCA) has seen an increasing incidence in recent years due to both lifestyle factors favoring its development and advancements in diagnostic methodologies.^2^^,^^3^ In the past decades, BTCs, which are usually diagnosed at an advanced stage, have been treated in palliative intention with chemotherapy typically consisting of gemcitabine/cisplatin, resulting in a median survival of less than one year, even when combined with immune checkpoint inhibitors lately.^4–6^ In recent years, however, various molecular alterations have been identified in BTC that can be addressed with targeted therapies.^7^

## Patient story

**Figure 1.**
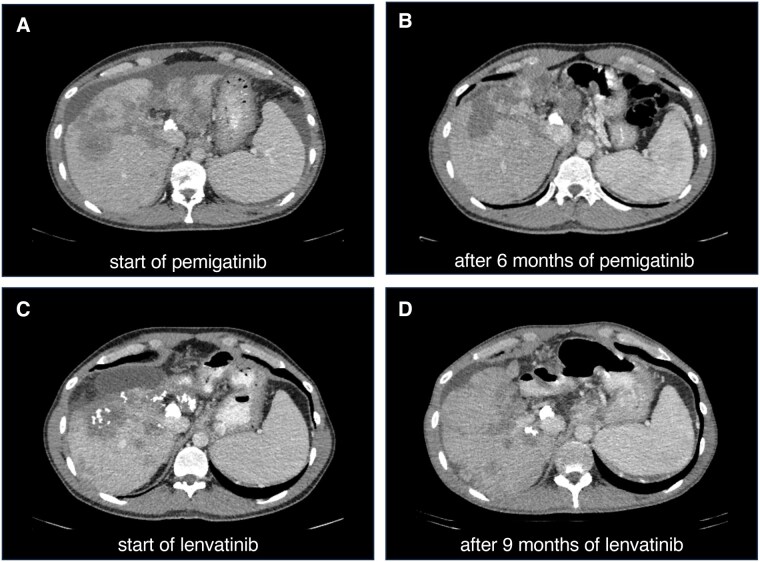
CT scans showing progressive disease under FOLFIRINOX therapy (A) and subsequent stable disease after 6 months of pemigatinib therapy (B). Following progression on pemigatinib and detection of an FGFR kinase point mutation, the tumor progressed further under futibatinib therapy (C). Disease remained stable after 9 months of lenvatinib treatment (D). Tumor response was evaluated according to RECIST 1.1 criteria.

A 41-year-old patient diagnosed with iCCA (cT2 cN1 cM0 G2) in September 2021 in Ukraine initially underwent approximately eight cycles of gemcitabine/cisplatin chemotherapy. Upon disease progression, therapy was switched to FOLFOX. After two cycles, treatment was interrupted in 2022 due to the outbreak of the war in Ukraine, prompting the patient to move to Germany. An initial staging CT scan at our clinic revealed progressive disease, with a growing primary tumor, extensive intrahepatic dissemination, and pulmonary metastases. At this point in the patient’s course, it was unclear whether the cancer was refractory to oxaliplatin. Therefore, platinum-based therapy was continued, and given the extensive liver involvement but preserved performance status, irinotecan was added (given as mFOLFIRINOX^8^). When the cancer continued to progress after three months of therapy, a tumor biopsy was performed in October 2022 for molecular pathological analysis (using the Illumina TSO500/TST170 panel). An oncogenic *FGFR2::BICC1* (F17B3) fusion was detected (Table 1). The patient was started on pemigatinib, which led to a significant tumor regression and was well-tolerated by the patient (Figure 1A and B). However, progression occurred after 14 months, so the tumor was re-biopsied in January 2024.

**Table 1.** Results of pathological, immunohistochemical, and molecular analyses of the two tumor biopsies.

	Biopsy I (13 months after diagnosis, post-FOLFIRINOX)	Biopsy II (28 months after diagnosis, post-pemigatinib)
**Pathology**		
**Report**	consistent with known cholangiocarcinoma	consistent with known cholangiocarcinoma
**Tumor cell content**	20%	15%
**Immunohistochemistry **		
**Line markers**	CK7 (strong), CK20 (moderate), CA19-9 (weak), TTF-1 (negative), NKX3 (negative), CDX2 (negative, HepPar1 (negative)	not repeated
**PD-L1 (22C3)**	TPS 0%, IC 0%, CPS 0	TPS 0%, IC <1%, CPS <1
**HER2**	not performed	0 (negative)
**Molecular pathology**		
**Used panel**	Illumina TSO500/TST170	Illumina TSO500 Plus/TST170
**TMB (Mut./Mb)**	2.4	1.6
**MSI**	3.45% (negative)	4.35% (negative)
**HRD score**	not performed	11 (negative)
**SNVs/Indels (allelic frequency)**	*MLH1* p.F88V (44%), *RUNX1T1* p.D7V (46%), *IRS2* p.G227R (63%), *IRF4* p.V128F (13%)	***FGFR2* p.E565A (6%),** *IL10* p.Y155H (4%), *PRKCI* p.R523L (6%), *MLH1* p.F88V (46%), *RUNX1T1* p.D7V (46%), *IRS2* p.G227R (46%)
**Copy number alterations**	none detected	none detected
**Splice variants**	none detected	none detected
**Translocations**	***FGFR2::BICC1* (F17B3)**	***FGFR2::BICC1* (F17B3)**

## Molecular Tumor Board

### Genotyping results and interpretation of the molecular results

In addition to its increasing use in clinical practice, liquid biopsy is gaining importance in this specific context for detecting resistance mutations, as it offers a less invasive approach.^9^ Because of a lack of reimbursement in Germany for liquid biopsy, this was not performed. However, its results would have been of interest, as it is likely to see wider application in the future. Given the accessibility of the tumor in our case, tissue biopsy was obtained. The Illumina TSO500 Plus/TST170 kit was used for DNA and RNA sequencing. In addition to the already known *FGFR2::BICC1* (F17B3) fusion, the point variant p. E565A was detected (specifically NM_000141: c.1694A>C; *FGFR2* isoform IIIc). This variant is referred to as p. E566A in *FGFR2* isoforms IIIb and IIIb C3 (transcripts NM_022970.3 and NM_001144913).^10^

Complementary immunohistochemistry and molecular pathology investigations were performed, but they did not yield findings that were of therapeutic relevance (Table 1).

### Functional and clinical significance of the specific mutation in the particular cancer

FGFR2 alterations are among the most significant, especially in iCCA, driving the increasing adoption of molecular genetic testing to identify such changes in this tumor type.^11^ In the presence of *FGFR2* alterations (mainly *FGFR2* fusions), selective FGFR-targeting tyrosine kinase inhibitors (TKIs) have recently been approved based on promising clinical data (EU and US approvals for pemigatinib and futibatinib), with their use now being endorsed by treatment guidelines from various specialist societies.^3^^,^^11^

Nevertheless, tumors often develop resistance to these TKIs in the long term, mirroring similar challenges observed in other malignancies treated with TKIs.^12^ Point mutations in the FGFR kinase domain seem to be primarily responsible for this resistance.^11^ The most frequently identified resistance-associated point mutations include the so-called “gatekeeper” mutation at amino acid V564, mutations in the molecular brake region (mostly N549, E565, and K641), and in the activation loop (such as K659).^9^^,^^12–14^

In the case described here, the detected p. E565A variant is located in the *FGFR2* kinase domain, specifically affecting one of multiple residues in the kinase hinge region referred to as “regulatory triad,” which mediate an autoinhibitory “molecular brake.”^14^

Recent studies have shown a polyclonal pattern of resistance mutation development, especially after treatment with reversible selective FGFR TKIs, further contributing to variable sensitivity to FGFR-targeted therapies.^15^^,^^16^

### Potential strategies to target the pathway and implications for clinical practice

Several reports have highlighted TKIs that may potentially overcome these resistance variants, although substantial clinical data are lacking to support these findings.^13^^,^^17^^,^^18^ For the p. E565A mutation detected in our patient, previous preclinical studies have described varying degrees of sustained therapeutic efficacy of the irreversible selective FGFR TKI futibatinib, with most studies describing at least partially preserved activity.^13^ Differences in experimental models, assay conditions, and co-mutations likely contribute to the general inconsistent sensitivity observed for kinase point mutations (overview in Table 2 for currently available literature regarding p. E565A). Clinical data for this mutation are, to our knowledge, not available.

**Table 2.** IC_50_ fold changes of approved FGFR inhibitors in p. E565A-mutated FGFR2 fusion-positive cell lines.

	Goyal et al. 2019	Goyal et al. 2023	Wu et al. 2024	Krook et al. 2020			Spahn et al. 2024
**Reference**	13	19	12	17			20
***FGFR2* fusion partner**	*PHGDH*	*TEL2*	*PHGDH*	*KIAA1598*			*AHCYL2*
**Cell line**	CCLP-1	Ba/F3	CCLP-1	NIH/3T3	293T	MMNK-1	NIH/3T3
**IC_50_ fold change p.E565A vs wild-type**							
**Pemigatinib**	8	8	35	–	–	–	–
**Futibatinib**	6	3	10	5	11	ambig.	29
**Erdafitinib**	–	1	18	20	65	15	–
**Lenvatinib**	–	–	–	–	–	–	3

Values rounded to the nearest whole number. –, not tested; ambig, ambiguous.

Recently, a study highlighted the efficacy of the (among others, FGFR-targeting) multikinase inhibitor lenvatinib in FGFR2-altered iCCA. Here, Spahn et al. demonstrated the effectiveness of lenvatinib in a patient with *FGFR2::BICC1* fusion and an additional p. N549K resistance-mediating kinase point mutation after disease progression on pemigatinib, and a partial response of nine months was achieved.^20^ Additionally, the authors showed retained activity of Lenvatinib in an *FGFR2::AHCYL2* plus p. E565A cell line model (Table 2).

Other studies examined the drug in the broader context of BTC. With an overall growing interest in combination therapies, studies of lenvatinib combined with immunotherapies (including nivolumab and pembrolizumab) have been conducted and showed an acceptable overall safety profile with some encouraging responses in advanced biliary tract cancer.^21–23^ Regrettably, there has been no further molecular workup of these tumors.

Several next-generation FGFR-targeting agents are being developed to address on-target resistance that commonly emerges after earlier FGFR inhibitors: Tinengotinib has shown signals of efficacy in FGFR2 fusion-positive CCA after prior FGFR-directed therapy (phase I/II), and is being evaluated in dedicated phase II and phase III trials in the FGFR inhibitor-refractory setting.^16^ Lirafugratinib is a highly FGFR2-selective inhibitor engineered to spare other FGFR paralogs and retain potency against prevalent resistance mutations, with early clinical responses in FGFR2-altered cholangiocarcinoma, including in patients previously exposed to FGFR inhibitors.^18^ However, outside of clinical trials, these inhibitors are not yet available for routine clinical use.

### Patient update

Despite the lack of clinical data and inconsistent preclinical data regarding preserved efficacy in *FGFR2* p. E565A mutated CCA, therapy was switched to futibatinib, mainly driven by its approval status and availability. In Germany, reimbursement for off-label drug use requires a formal approval process to the patient’s health insurance, which may take several weeks to months and is frequently subject to rejection. To avoid treatment delays, futibatinib was initiated promptly as an in-label option, while reimbursement for off-label lenvatinib was applied for in parallel.

After four months of treatment with futibatinib, there was immediate disease progression in the first staging examination (Figure 1C). Based on promising *in vitro* and *in vivo* data and following discussion in our molecular tumor board, in May 2024, treatment was switched to lenvatinib, which had already been approved for other indications. We chose to start lenvatinib at 24 mg daily (the dose recommended by EMA and FDA for the treatment of differentiated thyroid carcinoma; 12 mg daily is recommended for hepatocellular carcinoma) because of the patient’s young age, favorable performance status, preserved liver function, and absence of significant comorbidities. In comparison, Spahn et al. administered lenvatinib at 24 mg only as the peak dose, with lower doses used for most of the treatment period.

**Figure 2.**
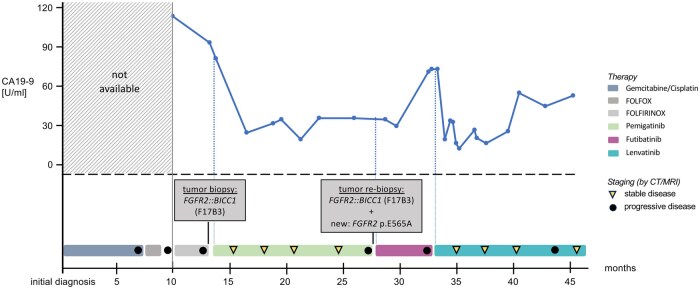
Individual treatment course of a patient with advanced iCCA with *FGFR2::BICC1* fusion. Corresponding RECIST response, CA19-9 levels are indicated as well as time points and liver biopsy results.

Remarkably, after the tumor had progressed on three different chemotherapy regimens and thereafter two selective FGFR targeting TKIs, lenvatinib therapy resulted in a slight regression of the tumor volume in the subsequent CT scan (formally classified as stable disease according to RECIST 1.1). This finding made further reimbursement by the health insurance company easier to obtain. In subsequent stagings, there was no marked radiographic response. Still, the disease remained stable, and treatment was continued in view of the overall clinical benefit. A small volume of perihepatic ascites was visible, which did not require drainage (Figure 1D). Tumor markers decreased after treatment initiation, and lenvatinib led to a remarkable progression-free survival of ten months (Figure 2).

At ten months, despite formal radiographic progression, Lenvatinib was continued in the absence of treatment alternatives and predominantly only small-sized progressive metastases with a stable primary tumor. A follow-up CT scan two months later revealed again stable findings without further growth of the metastases, and therapy has since continued (15 months in total to date).

The drug was well-tolerated overall. Transaminase levels (ALT, AST) increased initially (corresponding to Grade 3 in Common Terminology Criteria for Adverse Events v6.0) and declined following a dose reduction from 24 mg to 10 mg.

## Conclusion

FGFR-targeted therapy is critical in BTC, yet kinase mutations limit its long-term efficacy. Lenvatinib may provide a treatment option for patients with FGFR2 fusion-positive tumors resistant to FGFR-selective TKIs.

## Data Availability

The data underlying this article will be shared on reasonable request to the corresponding author.
